# A case of paroxysmal atrial fibrillation following low voltage electrocution

**DOI:** 10.1186/1756-0500-6-384

**Published:** 2013-09-27

**Authors:** Mitrakrishnan Rayno Navinan, Thambyaiah Kandeepan, Aruna Kulatunga

**Affiliations:** 1 National Hospital of Sri Lanka, Colombo, Sri Lanka

**Keywords:** Atrial fibrillation, Electrocution, Alternating current, Spontaneous resolution

## Abstract

**Background:**

Electrical injury may result in arrhythmias, however atrial fibrillation following low voltage electrocution is not a common occurrence.

**Case presentation:**

A 70-year-old South-Asian woman with no prior history of cardiovascular disease presented following an accidental low voltage electrocution with loss of consciousness. On initial assessment she was found to be in atrial fibrillation with a moderate to rapid ventricular rate. Troponin I and 2D echo were normal. Transient rise in markers of muscle damage were noted. The arrhythmia resolved spontaneously without active intervention.

**Conclusion:**

Loss of consciousness and the path of electrical conduction involving the heart may herald cardiac involvement following electrocution. Low voltage electrocution may cause cardiac insult. Conservative management may suffice in management of atrial fibrillation without cardiovascular compromise.

## Background

Electrical injury can cause a wide spectrum of cardiac complications ranging from myocardial necrosis with ventricular fibrillation [1] to less common arrhythmias. Atrial fibrillation however is not a common occurrence [2]. AF is known to occur with an increased incidence in advancing age, [3] and diabetes is a recognized risk factor for atrial fibrillation. However, commonly it is seen in association with ischaemic or structural heart disease [4]. Only a handful of electrocution induced new onset AF incidents have been reported [2,5-9], including AF precipitated by a lightning strike [10,11] and a case of a marine diver being electrocuted by a torpedo(electric) ray [12]. The mechanism leading to injury can be explained by the pathway taken by the transient circuit created when electrocution takes place, as it determines the tissue at risk. Blood, due to its high water and electrolyte content is low in resistance and acts as an excellent conductor of electricity [13]. High voltage electricity is usually considered the most dangerous [14]. However when the situation in which electrocution occurs is combined with a suggestive clinical picture [15], attention should not be diverted as even low voltage electrocution could cause cardiac insult [8]. With regards to management of atrial fibrillation following electrocution, lack of proper guidance and protocols has led to various modalities of treatment including DC cardioversion, pharmacological reversion or taking a conservative approach of watchful waiting for spontaneous resolution. Langford et al suggest that in the event of haemodynamic compromise to consider DC cardioversion advicing on medical cardioversion in all other situations [2] an approach also recommended by most guides in general for new onset AF management. However a systematic review regarding acute onset AF found that more than 50% revert to sinus rhythm within 24-48 hrs, especially when a precipitant is withdrawn [16]. We present a case of new onset atrial fibrillation that occurred following a low voltage electrocution which spontaneously reverted in a patient with no prior cardiovascular disease.

## Case presentation

**Figure 1:**
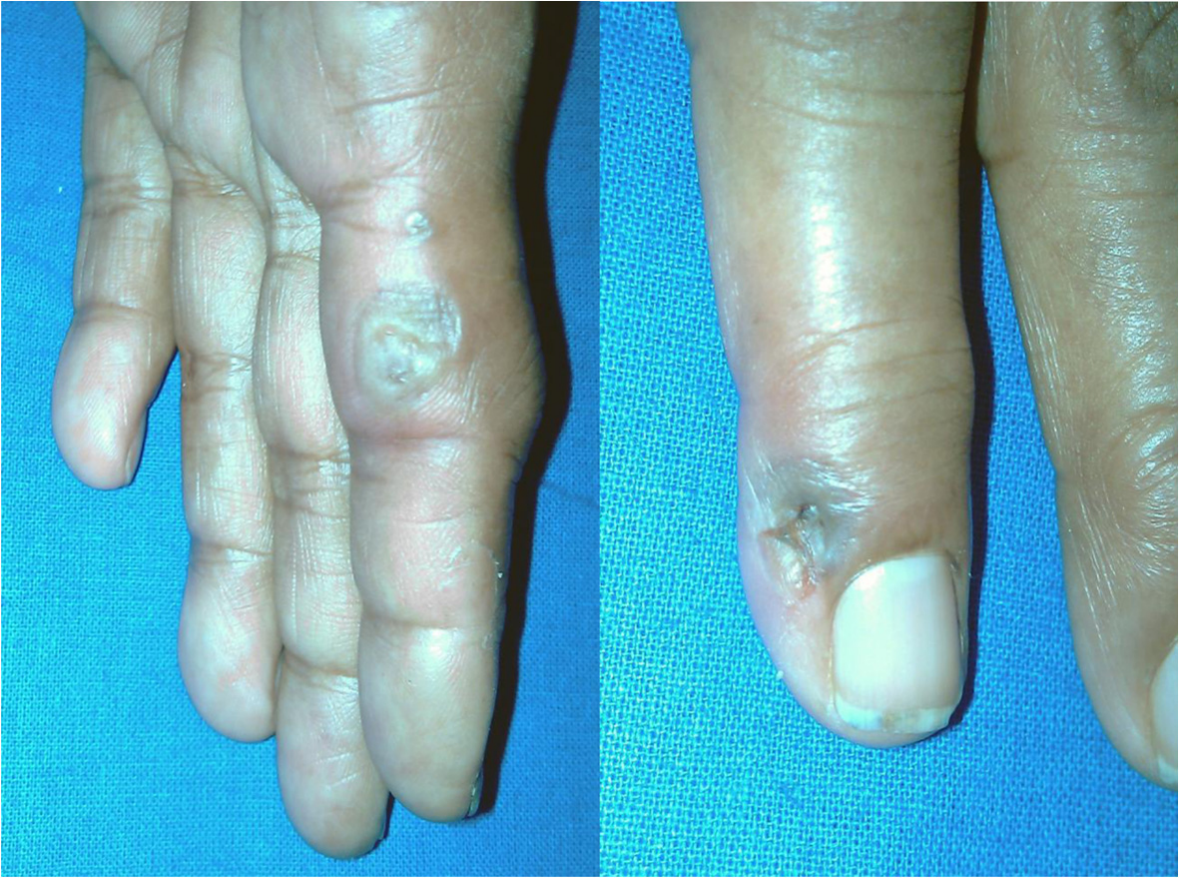
**The left image shows second degree burn injuries on the palmar aspect of the third phalanx of the left hand with blister formation.** The image on the right shows the little finger having similar injuries on the dorsal surface just below the nail.

**Figure 2:**
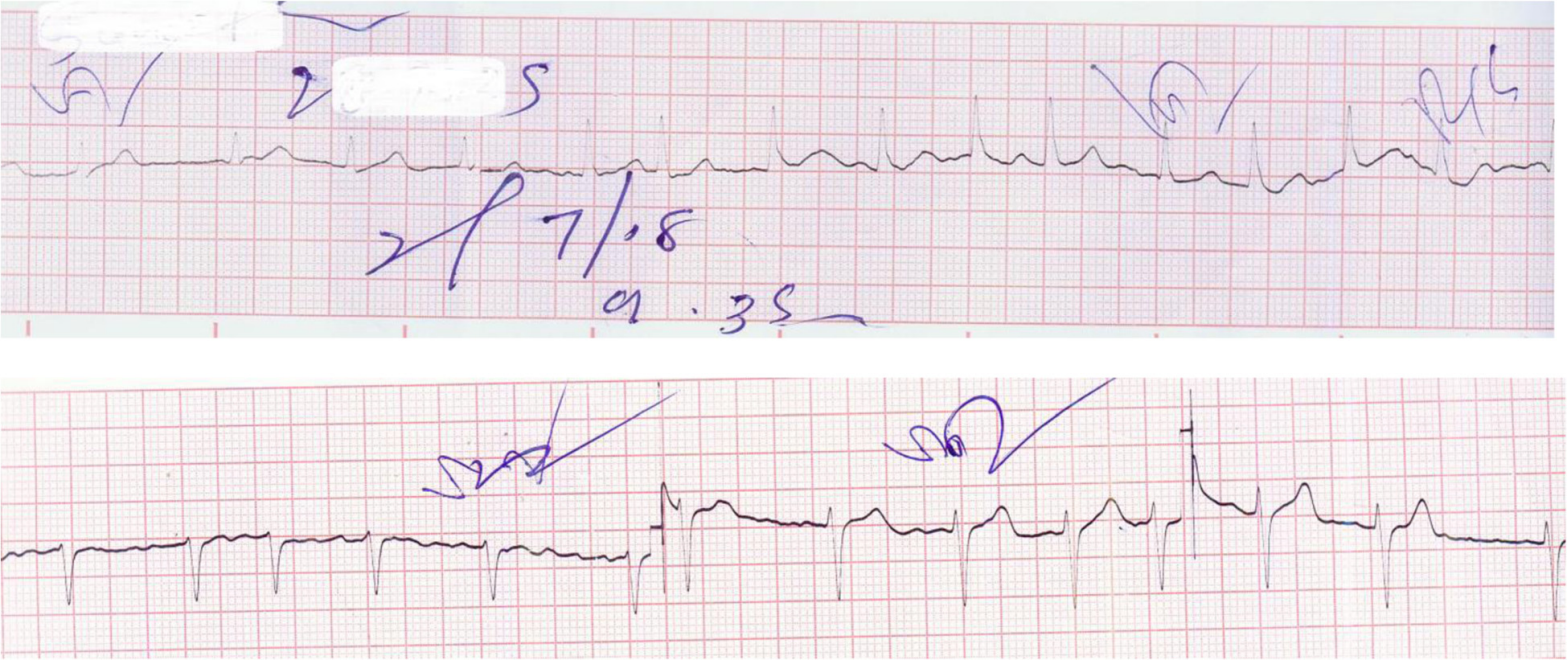
Preliminary ECG taken when patient was unconscious showing atrial fibrillation with a moderate to rapid ventricular rate.

A 70-year-old South-Asian woman who was known to be a diabetic with dyslipidaemia having good follow up care without a prior history of ischemic or hypertensive heart disease presented to us with transient loss of consciousness following an accidental electrocution. The electrocution occurred while attempting to change a household fluorescent bulb powered by low voltage alternating current of 220-240 V. Upon spontaneous recovery, the patient’s only complaint was left arm pain. Physical examination revealed a second degree burn injury of 1 cm diameter in both her hands indicating entrance and exit wounds (Figure 1). Systemic examinations of the neurological and respiratory systems did not reveal notable anomalies. Examination of the cardiovascular system revealed an irregularly irregular pulse with a moderate to rapid rate. Patient was haemodynamically stable with no overt features of heart failure and the rest of the cardiovascular examination was normal. Electrocardiogram revealed atrial fibrillation with a moderate to rapid ventricular rate (115-150 beats per minute) without any ischemic features (Figure 2). Cardiac and muscle markers done immediately demonstrated elevated levels of Myoglobin-294.5 ng/mL, CK-MB-17.4 ng/mL and a normal Troponin I value of 0.2 ng/mL (Reference values: Myoglobin 0-80 ng/mL, CK-MB 0-5.0 ng/mL, Troponin I 0-0.5 ng/mL). Eight hours post incident, markers were rechecked and both Myoglobin and CK-MB had normalized and Troponin remained within normal limits. Serum sodium, potassium and thyroid function tests were also normal. A 2D echocardiogram gave a normal study and identified no prior indication of ischemic or hypertensive heart disease.

**Figure 3:**
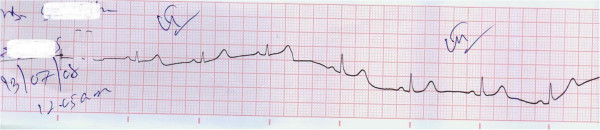
ECG taken following spontaneous reversion to sinus rhythm.

**Figure 4:**
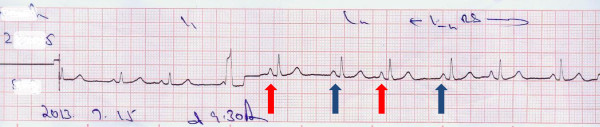
ECG taken on review one week after spontaneous reversion demonstrates a ventricular premature complex with two different atrial morphologies (shown in red and blue arrows) suggesting atrial premature complexes (denoted by red arrows) in a supraventricular bigeminy pattern.

High dependency unit management was instituted with continuous electrocardiographic observation. In the absence of hemodynamic compromise, a rapid ventricular rate was the only feature which was controlled with atenolol. The atrial fibrillation reverted to sinus rhythm within 6 hours spontaneously (Figure 3). Patient was kept under electrocardiographic monitoring on day one and repeated ECG’s were taken and assessed on day two. Upon discharge patient was in sinus rhythm. She was reviewed one week later with an electrocardiogram which demonstrated atrial premature complexes (Figure 4).

Our patient had an electrocution precipitated first episode of atrial fibrillation. The short duration, spontaneous resolution coupled with an absence of a prior cardiovascular comorbid history and lack of structural anomalies favored electrocution induced AF. The electrocardiogram taken on review did demonstrate premature atrial complexes. As this phenomenon is yet to be reported as occurring de-novo following electrocution we could hypothesize that our patient already had atrial premature complexes from the outset. It is recognized that APCs increase the risk of arrhythmias both supraventricular and ventricular in origin but is commonly associated with paroxysmal atrial fibrillation [17-19]. Although associated with structural heart disease and chemical exposure such as alcohol and caffeine, APCs can also be an incidental and normal finding in otherwise healthy individuals [20]. Therefore we could postulate that in the context of pre-existent APCs, the accidental electrocution may have been the necessary trigger to precipitate a PAF.

Commonly, high voltage electrocution is thought to precipitate cardiac abnormalities with a high incidence [14]. Our subject developed AF from a low voltage (220-240 V) insult, an observation previously noted as well [8]. In our patient the heart was naturally in the path of the electrical current as both the entrance and exit wounds were located in each hand. A study done by Purdue et al suggested that loss of consciousness following an electrical insult is an indication for observation by electrocardiographic monitoring [15] a conclusion possibly reaffirmed by our case. Spontaneous resolution of electrocution induced arrhythmia has also been previously recorded [5,10,21-23]. Our case also proved, diligent watchful expectancy with rate control can be an alternate method of management.

## Conclusions

This case demonstrates that low voltage electrical insults should not be taken lightly. When features suggesting possible cardiac involvement are present such as loss of consciousness and when the path of least resistance of electricity includes the heart, a high suspicion of a possible cardiac insult should be considered, investigated and appropriately monitored. Pre-existent electrical anomaly such as atrial premature complexes may increase the risk of developing paroxysmal atrial fibrillation following electrocution. Management need not always be interventional, as rate control when relevant with watchful waiting with electrocardiographic monitoring can be an effective and less traumatic method of management.

## Consent

Written informed consent was obtained from the patient for publication of this case report and accompanying images. A copy of the written consent is available for review by the Editor-in-Chief of this journal.

## Abbreviations

AF: Atrial fibrillation; DC: Direct current; PAF: Paroxysmal atrial fibrillation; APCs: Atrial premature complexes.

## Competing interests

The authors declare that they have no competing interests.

## Authors’ contributions

MRN, AK, TK diagnosed the clinical scenario. MRN & AK researched & drafted the documented. All authors provided care for the patient. All authors read and approved the final manuscript.

## Authors’ information

MRN is a registrar of medicine at the National Hospital of Sri Lanka, Colombo.

AK is a Consultant physician in acute medicine at the National Hospital of Sri Lanka, Colombo.

TK is a senior registrar in medicine at the National Hospital of Sri Lanka, Colombo.
